# HEXACO Personality Factors and their Associations with Facebook use and Facebook Network Characteristics

**DOI:** 10.1177/00332941231176403

**Published:** 2023-05-26

**Authors:** Riana M Brown, Sam GB Roberts, Thomas V Pollet

**Affiliations:** Department of Psychology, 5894 New York University, USA; School of Psychology, 4589 Liverpool John Moores University, UK; Department of Psychology, 5995 Northumbria University, UK

**Keywords:** personality, social networks, network structures, HEXACO, Facebook

## Abstract

Personality factors affect the properties of ‘offline’ social networks, but how they are associated with the structural properties of online networks is still unclear. We investigated how the six HEXACO personality factors (Honesty-Humility, Emotionality, Extraversion, Agreeableness, Conscientiousness and Openness to Experience) relate to Facebook use and three objectively measured Facebook network characteristics - network size, density, and number of clusters. Participants (*n* = 107, mean age = 20.6, 66% female) extracted their Facebook networks using the GetNet app, completed the 60-item HEXACO questionnaire and the Facebook Usage Questionnaire. Users high in Openness to Experience spent less time on Facebook. Extraversion was positively associated with network size (number of Facebook Friends). These findings suggest that some personality factors are associated with Facebook use and the size of Facebook networks, and that personality is an important influence on both online and offline sociality.

## Introduction

Having a strong and supportive network of social relationships is a key factor in both physical health and psychological well-being (Baumeister & Leary, 1995a, 1995b; Hawkley, 2022; Holt-Lunstad et al., 2010; Portela et al., 2013). Personality characteristics have been linked with specific characteristics of these ‘offline’ social networks such as network size and emotional closeness to network members (Molho et al., 2016; Pollet et al., 2011; Rollings et al., 2022; Selden & Goodie, 2018). With the increasing popularity of social networking sites, research has focused on how personality characteristics are associated with individual differences in online sociality (Bowden-Green et al., 2020; Huang et al., 2018; Ross et al., 2009). Facebook remains the largest social networking site, with 1.96 billion daily active users (Dixon, 2022a), and a range of personality characteristics have been associated with Facebook use (Bowden-Green et al., 2020; Ross et al., 2009).

However, in addition to the size of social networks, the structural characteristics of social networks have important consequences. Denser networks are those with more connections between members of the social network, and density in offline networks is associated with higher levels of social support (Bell, 1991) and lower feelings of loneliness (Stokes, 1985). To date, there has been very limited research systematically examining how the full range of personality characteristics are associated with the size and structure of online social networks (Lönnqvist et al., 2014; Noë et al., 2016). In this study, we examined how objectively measured Facebook network size and structure were associated with the six HEXACO factors of personality (Ashton & Lee, 2007, 2020) - Honesty-Humility (H), Emotionality (E), Extraversion (X), Agreeableness (A), Conscientiousness (C) and Openness to Experience (O).

### Personality and Offline Social Networks

Many personality traits relate in some way to sociality and are relatively stable over time (Ashton & Lee, 2007; Costa & McCrae, 1992). Thus, these traits may influence the size and structure of people’s social networks, consisting of the ties people have to family and friends, and the ties between these family members and friends (for a review, see Selden & Goodie, 2018). Extraversion is associated with both larger network size (Pollet et al., 2011) and faster addition of new network members (Feiler & Kleinbaum, 2015; Wagner et al., 2014; Zhu et al., 2013). However, one study found that the relationship between extraversion and network size disappeared after controlling for age (Roberts et al., 2008). Both Agreeableness and Openness to Experience are associated with a larger network size (Wagner et al., 2014; Zhu et al., 2013), whilst Conscientiousness is associated with more family members in the network (Doeven-Eggens et al., 2008; Wagner et al., 2014). Lastly, despite Neuroticism being characterized by negative affect and social anxiety (Costa & McCrae, 1992), this personality trait does not seem to be strongly associated with either network size or composition, although it is associated with lower levels of emotional closeness to network members (Wagner et al., 2014; Zhu et al., 2013)

Whilst most studies have used the Five-factor model of personality (Selden & Goodie, 2018), a study using the six factor HEXACO personality model showed that Extraversion, Openness to Experience, and Emotionality were positively related to the size of the support group (the small number of very close relationships in the network), whilst Honesty-Humility was associated with the level of emotional closeness to members of the sympathy group (a slightly larger grouping of close friends and family; Molho et al., 2016).

In terms of the link between ‘offline’ and ‘online’ sociality, Facebook profiles accurately reflect the self-reported personality of Facebook users (Back et al., 2010; Ross et al., 2009) and Facebook is used primarily to maintain and strengthen relationships that also exist offline (Burke & Kraut, 2016; Ellison et al., 2014; Subrahmanyam et al., 2008; Sutcliffe et al., 2018, 2022). Thus, if Facebook networks at least partially reflect offline sociality, it may be expected that there would be a relationship between personality and the size and structure of Facebook networks.

Further, associations between personality traits and the specific affordances of social networking sites can be understood within the situation, trait and outcome activation framework (De Vries et al., 2016). The situation activation mechanism proposes that people consciously or unconsciously seek out situations that fit their personality traits. Social networking sites enable social interaction with a large number of different users, and thus it may be expected that, for example, Extraverts would spend more time on social networking sites than Introverts, since the core of Extraversion is a tendency to behave in ways that attract social attention (Ashton et al., 2002). In terms of trait activation, social networking sites offer many affordances that may increase the likelihood of specific personality traits being expressed. For example, Extraverts may express their need for social attention by having a larger network of Facebook Friends and posting more content. Finally, the outcomes of expression of personality traits on social networking sites may vary across different traits – for example, Extraverts may get more positive feedback if they post a lot of content, and thus may find using social networking sites more rewarding than Introverts, leading them to spend more time on the site.

### Personality and Online Social Networks

Research has explored the association between personality and online sociality, with a particular focus on the Extraversion-Introversion continuum (see Bowden-Green et al., 2020 for a review). Those high on Extraversion have a higher number of Facebook Friends,^1^ are members of more Facebook groups, use Facebook more, and comment more frequently on Facebook (Amichai-Hamburger & Vinitzky, 2010; Gosling et al., 2011; Lönnqvist et al., 2014; Noë et al., 2016; Shen et al., 2015). These findings support the ‘rich-get-richer’ hypothesis, whereby users with an outgoing and sociable personality gain most from the ease of communication afforded by social networking sites. Whilst most research has found a positive relationship between extraversion and Facebook use (Bowden-Green et al., 2020), Ross et al. (2009) did not find that Extraversion was significantly related to number of Facebook Friends or time spent online.

Researchers have also explored the association between other personality traits and Facebook use. People higher in Openness to Experience have a greater tendency to be sociable through Facebook (Amichai-Hamburger & Vinitzky, 2010; Ross et al., 2009) and change their profile picture more often (Gosling et al., 2011). Individuals who are higher on Agreeableness do not tend to have more Facebook Friends, contrary to the authors’ hypotheses (Amichai-Hamburger & Vinitzky, 2010; Eşkisu et al., 2017; Lönnqvist et al., 2014; Ross et al., 2009). Amichai-Hamburger and Vinitzky (2010) found that those high on Conscientiousness have more Facebook Friends, but upload fewer photos to the site. Gosling et al. (2011) found those low on Conscientiousness spent more time on Facebook, whilst other studies have not found a significant association between Conscientiousness and Facebook use (Eşkisu et al., 2017; Lönnqvist et al., 2014; Ross et al., 2009). Overall, there has been a mixed pattern of results relating personality to the number of Facebook Friends and other characteristics of Facebook use. In line with personality research in general (Butcher et al., 1995; Hunsley & Meyer, 2003), the effect size of personality factors on Facebook use tends to be small to medium (Bowden-Green et al., 2020; Ross et al., 2009). Thus, some of the variance in results may be due to different sample populations, different measures of Facebook use (e.g., self-report vs. data gathered from Facebook directly) and different measures of personality (e.g., dichotomous vs. continuous measures of personality factors).

More recently, researchers have used data gathered from Facebook directly to examine how structural network properties are associated with personality. Lönnqvist et al. (2014) examined the size of Facebook networks and also network density – the proportion of all possible friendship ties that are present in the participants’ Facebook networks (i.e. whether the participants Facebook Friends are also Friends with each other). High levels of Extraversion were associated with a larger network size, but lower network density. However, larger networks were also less dense, and when network size was controlled for, the relationship between extraversion and density was no longer significant. Further, high levels of Openness to Experience were associated with lower density only for men (Lönnqvist et al., 2014). Noë et al. (2016) replicated this gender specific effect for Openness to Experience and found Extraversion was also positively related to network size, whilst Neuroticism was negatively related to network size. Even after controlling for network size, those high in Extraversion had less dense networks, although the large sample size (*n* = 9,569 for this analysis) means results can be statistically significant even with very small effect sizes. Apart from these two studies, to our knowledge, other studies have yet to examine the relationship between personality and the structural characteristics of Facebook networks.

In this study, we focus on three Facebook network characteristics. The network size is defined as the number of Facebook Friends. The network density is the proportion of all possible Facebook Friendship ties that are present in the participants Facebook network (Bastian et al., 2009). Finally, the number of clusters in the network (sub-groups of closely connected people) provides an indication of whether the network consists of a large number of separate individuals or is made up cohesive groups of Facebook Friends (Bastian et al., 2009). Research on offline social networks has demonstrated that personality factors are associated with not just the size of social networks, but also these structural characteristics of networks (for a review, see Selden & Goodie, 2018). Higher levels of Extraversion are associated with more connections specifically between strong ties (Kalish & Robins, 2006), but does not appear to be related to overall density in the network (Kalish & Robins, 2006; Stokes, 1985; Zell et al., 2014). In contrast, Neuroticism is not related to the overall density of the network but is related to fewer connections between strong ties (Kalish & Robins, 2006; Stokes, 1985). Whilst studies of the other personality factors and network position and structure exist, particularly in workplace settings (Selden & Goodie, 2018), less is known about how these other personality factors affect density or number of clusters in the network. Given the well-established associations between personality characteristics and both online and offline sociality (Bowden-Green et al., 2020; Lönnqvist et al., 2014; Selden & Goodie, 2018), this study explored how the three Facebook network characteristics (size, density, number of clusters) were associated with personality factors.

### Study Rationale and Predictions

This study adds to existing research in this area in two key ways. First, we extracted information on the size and structure of Facebook networks directly from participants’ Facebook accounts, rather than relying on self-reported Facebook network size as in some previous research (Eşkisu et al., 2017; Marshall et al., 2015). Having this detailed network data allows us to examine the associations between personality and network structures (density and number of clusters), rather than just focusing on network size or Facebook usage. Second, instead of assessing Extraversion exclusively, or the Big Five, we used the HEXACO personality model (Ashton & Lee, 2007, 2020) to examine how the whole range of personality factors are associated with Facebook use, network size and network structure. There is strong cross-cultural support for an alternative six-dimensional personality model which reorganizes the personality factors from the Five-factor model and adds a new dimension, Honesty-Humility (Ashton et al., 2014; Ashton & Lee, 2007, 2020). Specifically, we make the following predictions for how the six HEXACO personality factors are related to Facebook networks:

(1) People who are high on Extraversion are characterized as very sociable (Selden & Goodie, 2018) and may use Facebook as another outlet for this sociality (Bowden-Green et al., 2020; Lönnqvist et al., 2014; Noë et al., 2016; Shen et al., 2015). Extraverts tend to have larger networks (Pollet et al., 2011; Selden & Goodie, 2018) and generally there is a negative relationship between network size and density (Faust, 2006; Lönnqvist et al., 2014). We thus expect Extraverts to (a) spend more time on Facebook, (b) view Facebook as part of their everyday activity, (c) have a larger network size, (d) have lower network density, (e) have more clusters.
(2) Those who are high on Openness to Experience are more curious and seek exposure to others who are different from themselves (Ashton & Lee, 2007). Therefore, we predict that people who score higher on Openness to Experience will have (a) low network density, reflecting Friends from different places or contexts who do not have links to other Friends, (b) have more clusters as they seek out a diverse range of friendship groups.
(3) People who are high on Agreeableness engage in caring and meaningful offline relationships (Selden & Goodie, 2018; Wagner et al., 2014; Zhu et al., 2013). We therefore predict participants high on Agreeableness will have (a) a larger network, (b) lower network density, (c) fewer clusters, because they seek more meaningful friendship groups.
(4) High Conscientiousness involves high-target orientation, fulfillment of obligations and strong management of time (Ashton & Lee, 2007; Costa & McCrae, 1992). Thus, we expect individuals with high Conscientiousness will (a) spend less time on Facebook, (b) view Facebook less as a part of their everyday activity, (c) have more Facebook friends, (d) have a low network density, because they can manage relationships more easily without having to rely on Friends who know each other.
(5) For exploratory purposes, we also make predictions for Emotionality. Individuals with larger offline networks tend to be less emotionally close to the people in their network (Binder et al., 2012; Pollet et al., 2011). Those high on emotionality have a higher need for emotional support and close social relationships (Ashton et al., 2014), and therefore we expect them to have (a) lower network size, (b) higher network density, reflecting a more tight knit network with Friends who know each other, (c) fewer clusters, due to the focus on a fewer, closer relationships
(6) Lastly, we explored whether those high on Honesty-Humility, who do not have a strong desire to pursue social status (Ashton et al., 2014), will have (a) a smaller network size.

We controlled for age, sex and relationship status in our analyses because previous research has found that these variables are related to both personality and properties of social networks (Gosling et al., 2011; Lönnqvist et al., 2014; Pollet et al., 2011; Roberts et al., 2008).

## Method

### Participants

Initially, 110 participants provided informed consent and took part in the study. This is sufficient to be able to detect a weak to moderate effect size (*r* = .263; assumed power = .8, based on a two-tailed correlation test; (Faul et al., 2007)). Three of the participants were excluded from the final analysis - two participants did not follow the instructions correctly and one participant was an extreme outlier due to the number of Facebook Friends (four standard deviations above the mean). The final sample consisted of 107 participants (71 women, 36 men) between the ages of 18–32 years (*M* = 20.6 years, *SD* = 2.71 years) and were all students at a large European university. The majority of participants were Dutch (*N* = 95), with 10 other nationalities reported (*N* = 12). In terms of relationship status, 44 of the participants reported having a partner (were married or in a committed relationship). Participants received either 5 euros or 45 minutes worth of study credits for completing the study. All participants were required to have a Facebook account.

### Procedure and Measures

Participants completed the study in individual cubicles in a laboratory at the university. We informed participants that the study intends to extract their Facebook network data using the *GetNet* app, a modified version of *Netvizz* (Adamic, 2015; Rieder, 2013). We asked the participants to log on to their Facebook profiles and agree to download the *GetNet* app. We then transferred the *GetNet* files to the open-source network analysis software Gephi (Bastian et al., 2009) for calculation of the network measures. The *network size* is the number of Facebook Friends in the users’ network. The *network density* was computed by dividing the number of connections between Facebook Friends in the network (i.e. if they were Friends with each other) by the number of all possible connections. Density values can range from 0 (none of the individuals in the participants’ network are Friends with each other) to 1 (all the individuals in the participants’ network are also Friends with each other). Finally, the *number of clusters* within the network represents the number of tightly connected sub-groups (cliques) consisting of at least three individuals and was calculated using the Louvain method within Gephi (Blondel et al., 2008).

Participants then completed a series of questionnaires. We used two questions from the Facebook questionnaire (Ross et al., 2009) to measure Facebook usage: “On average, approximately how much time per day do you spend on Facebook?” (1 = *10 minutes or less* to 6 = *three or plus hours*) and “Facebook is a part of my everyday activity” (1 = *strongly disagree* to 5 = *strongly agree*). These items were used to create the variables *Facebook time* and *Facebook everyday*^2^*.* The participants also completed the 60-item HEXACO Personality Inventory, which contains 10 items for each of the six personality factors (Ashton & Lee, 2009). After reverse scoring, we calculated a mean score for each factor, with high scores indicating higher levels of that factor. In our sample the Reliability coefficients ranged from Cronbach’s alpha of .69–.81 (Honesty-Humility, α = .77; Emotionality, α = .81; Extraversion, α = .81; Agreeableness, α = .71; Conscientiousness, α = .69, Openness to Experience, α = .72). Whilst there is no definitive agreement on how to interpret Cronbach’s alpha values (Cortina, 1993; Field, 2013; Streiner, 2003), all the personality factors were around or above the commonly used 0.7 alpha level for acceptable reliability.

The participants provided demographic information relating to their gender, age, level of education, nationality, native language, and current relationship status. The participants also completed further questionnaires as part of a broader study. These were the UCLA Loneliness Scale (Russell, 1996), a paper list of their top 20 friends, and questions relating to the ‘inner’ layers of their online and offline social networks (Binder et al., 2012; Buys & Larson, 1979). The association between loneliness and Facebook networks in this sample has been examined in previous research (Brown et al., 2021). Brown et al. (2021) did not include any analysis of the association between personality traits and Facebook networks. The average duration of the survey was 45 minutes.^2^

In terms of ethical issues, the participants did not share their Facebook login details with the researchers, but instead logged in themselves to their own Facebook account to download the *GetNet* app and extract the data. Once the data was extracted, it was transferred to Gephi, where social network metrics were calculated by the researchers and added to our dataset. After the calculations of key social network metrics (size, density, number of clusters), the social network data were removed. Via this method, we obtained Facebook social network metrics without storing any personally identifying information about the participants or their Facebook friends, thus ensuring the anonymity of the Facebook network data. Similarly, the questionnaires were completed anonymously. Ethical approval was granted for this study by the local ethics committee at VU Amsterdam (ref: VCWE-2015–003).

### Design and Statistical Analysis

First, we present descriptive statistics and bivariate correlations. We then present results from a series of hierarchical Ordinary Least Squares (OLS) regressions for Facebook usage and for Facebook network characteristics. There were six dependent variables in six separate regression models: Facebook time, Facebook everyday, Network size, Log Network size, Network density and Number of clusters. For each of these six regression models, we first included all six HEXACO personality factors as predictors, because these are the key variables of interest. Next, we ran the model retaining only the marginally significant and significant personality predictors (*p < .*10) and added the control variables in the following predetermined order: gender, age, nationality, and relationship status. For the analysis of network density, we also controlled for network size, as previous research has demonstrated a strong negative relationship between network size and density (Faust, 2006; Lönnqvist et al., 2014). We kept only significant control variables in our final models (*p* < .05). Additionally, we also computed a logarithmic transformation of network size to correct for skewness regarding network size. In order to ensure the robustness of our results, in the Electronic Supplementary Information (ESM) we report the results of the OLS regression models using a bootstrap procedure (Bias Corrected and Accelerated (BcA) bootstrap with 10,000 samples) (Davison & Hinkley, 1997; Efron, 1987). In the ESM, we also report robust standard errors (correcting for heteroscedasticity and autocorrelation; Freedman, 2006; Huber, 1967; Huber, 2011; White, 1982). We used R 4.0.2 for all analyses (R Development Core Team, 2008). The data, code and ESM are available on the Open Science Framework, OSF (https://osf.io/4kjfp/).

## Results

### Descriptive Statistics and Bivariate Correlations

The mean for Facebook time was 2.98 and for Facebook everyday was 3.65, indicating the participants spend an average of 30 minutes–2 hours on Facebook per day, and agreed that Facebook was part of their everyday activities (Table 1). The mean network size (number of Facebook Friends) was 394 (range 29–1095), and mean density was .10 (range .02–.24), meaning that 10% of the possible connections between the Facebook Friends in the participants’ networks were present (Table 1). The mean number of network clusters was 16 (range 5–49), so each participant was on average part of 16 tightly connected clusters of Facebook Friends. There was a large range in the number of clusters between participants, so some participants had many tightly connected clusters of Friends in their Facebook networks, whilst other participants had very few.

**Table 1.** Means, Standard Deviations, and Pearson Correlations with 95% Confidence Intervals.

Variable	*M*	*SD*	1	2	3	4	5	6	7	8	9	10	11	12	13	14
1. Facebook time	2.98	1.16														
2. Facebook everyday	3.65	1.07	.53** [.37, .65]													
3. Network size	394.08	225.84	.31** [.12, .47]	.29** [.11, .46]												
4. Density	0.10	0.04	−.17 [-.35, .02]	−.16 [-.34, .03]	−.26** [-.43, −.08]											
5. Clusters	16.38	9.25	.19* [.00, .37]	.15 [-.04, .33]	.48** [.32, .61]	−.59** [-.70, −.45]										
6. Gender	0.66	0.47	.18 [−.01, .36]	.16 [−.03, .34]	.04 [−.15, .23]	.14 [−.05, .32]	−.22* [-.39, −.03]									
7. Age	20.64	2.71	−.18 [-.36, .01]	−.32** [-.48, −.14]	−.28** [-.45, −.10]	−.35** [-.51, −.18]	.19* [.00, .37]	−.32** [-.48, −.14]								
8. Nationality	0.11	0.32	.21* [.02, .39]	.06 [-.13, .25]	.13 [-.07, .31]	−.18 [-.36, .01]	.11 [-.08, .30]	−.12 [-.31, .07]	.37** [.19, .52]							
9. Relationship	0.41	0.49	−.18 [−.36, .01]	.02 [−.17, .21]	.09 [−.10, .28]	−.01 [−.20, .18]	−.08 [−.27, .11]	−.05 [−.24, .14]	.01 [−.18, .20]	−.12 [−.30, .08]						
10. Honesty-humility	2.79	0.35	.09 [−.10, .28]	.12 [−.07, .30]	.04 [−.15, .23]	−.03 [−.21, .17]	−.02 [−.21, .17]	.32** .14, .48]	−.02 [−.20, .18]	.08 [−.11, .27]	−.05 [−.24, .14]					
11. Emotionality	2.91	0.63	.12 [−.07, .30]	.14 [−.05, .33]	.12 [-.07, .30]	.05 [−.14, .24]	−.16 [−.34, .03]	.63** [.50, .73]	−.23* [−.40, −.04]	.00 [−.19, .19]	.15 [−.05, .33]	.06 [−.13, .25]				
12. Extraversion	3.07	0.56	.19 [−.00, .36]	.18 [−.02, .35]	.39** [.22, .54]	−.20* [−.38, −.01]	.24* [.05, .41]	−.02 [−.20, .17]	−.11 [−.29, .08]	.10 [−.09, .28]	−.00 [−.19, .19]	.10 [−.10, .28]	−.17 [−.35, .02]			
13. Agreeableness	2.67	0.53	−.03 [−.22, .16]	−.02 [−.21, .17]	−.03 [−.22, .16]	.08 [−.12, .26]	−.09 [−.28, .10]	.05 [−.14, .23]	−.07 [−.26, .12]	−.18 [−.35, .01]	−.03 [−.21, .16]	.29** [.10, .45]	−.07 [−.26, .12]	−.02 [−.21, .17]		
14. Conscientiousness	2.93	0.49	.03 [−.16, .22]	.08 [−.11, .26]	.13 [−.06, .31]	−.00 [−.19, .19]	.13 [−.07, .31]	.17 [−.02, .35]	−.03 [−.22, .16]	.12 [−.07, .30]	.01 [−.18, .20]	.03 [−.16, .22]	.12 [−.07, .31]	.13 [−.06, .31]	−.03 [−.22, .16]	
15. Openness	2.88	0.59	−.22* [−.39, −.03]	−.17 [−.35, .02]	−.12 [−.31, .07]	−.15 [−.33, .05]	.10 [−.10, .28]	−.31** [−.47, −.12]	.20* [.01, .38]	.13 [−.06, .31]	−.11 [−.30, .08]	.05 [−.14, .24]	−.38** [−.53, −.21]	.04 [−.15, .23]	.04 [−.16, .22]	−.05 [−.23, .14]

*Note. M* and *SD* are used to represent mean and standard deviation, respectively. Values in square brackets indicate the 95% confidence interval for each correlation. The confidence interval is a plausible range of population correlations that could have caused the sample correlation (Cumming, 2014). * indicates *p* < .05. ** indicates *p* < .01. Coding for categorical variables: Gender (0 = Male, 1 = Female), Nationality (0 = Dutch, 1 = Other nationality). Relationship status (0 = Not in a relationship, 1 = In a relationship).

### Demographics and Network Characteristics

We first examined how Facebook usage and network characteristics were associated with the demographic characteristics of the participants (gender, age, nationality and relationship status). We examined the pairwise relationships between these variables using Pearson correlations and Welch t-tests (Table 1). We also used hierarchical regression analysis to examine which demographic characteristics were significantly associated with the Facebook use and network characteristics, whilst controlling for the other demographic characteristics. These regression results are available on the OSF page (https://osf.io/4kjfp/).

The demographic characteristic most strongly associated with the Facebook variables was age. Younger participants were significantly more likely to agree that Facebook was part of their everyday activity, had significantly more Facebook Friends, a significantly lower density network, and a significantly larger number of clusters (Table 1). Males had significantly more clusters than females (Table 1). A Welch *t*-test also found a significant difference in the number of clusters between males and females (*t* (60.47) = 2.14, *p* = .037). Finally, non-Dutch participants reported spending significantly more time on Facebook as compared to Dutch participants (Table 1). A Welch *t*-test found a marginally significant difference (*t* (13.43) = −2.07, *p* = .059) for Facebook time and nationality.

### Personality and Facebook Usage Variables

**Figure 1.**
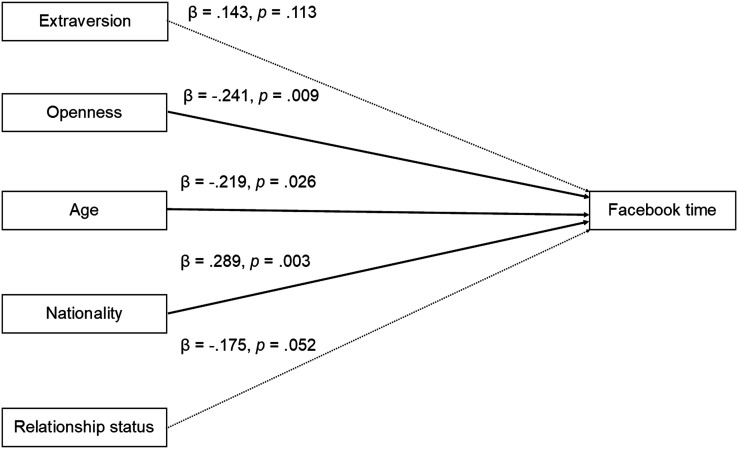
Summary of regression predicting self-reported time spent on Facebook from personality and demographic variables. Figure shows final model (*F* (5, 102) = 5.51, *p* < .01, adjusted *R*^2^ = .17). Significant predictors of Facebook time (Openness, Age, Nationality) indicated with solid lines; non-significant predictors of Facebook time (Extraversion, Relationship status) indicated with dashed lines. Standardised coefficients (*β*) and *p* values displayed for each predictor. Nationality (0 = Dutch, 1 = other nationality); Relationship status (0 = Not in a relationship, 1 = In a relationship).

In the regression models, there was a significant positive association between Extraversion and Facebook time, initially supporting hypothesis 1(a) (ESM Table 1). There was also a significant negative relationship between Openness to Experience and Facebook time. No other personality variables were significantly related to time spent on Facebook (all *p* > .37). The final Model 6 was significant (*F* (5, 102) = 5.51, *p* < .01), accounting for 17% of the variance in time spent per day on Facebook (*R*^2^ = .21, adjusted *R*^2^ = .17). This final Model 6 contained a statistically significant effect of Openness (β = −.24, *p* < .01) but not of extraversion (β = .14, *p* = .11, Table 2, Figure 1). Thus, overall participants high in Openness reported spending significantly less time on Facebook than those low in Openness.

**Table 2.** Hierarchical Regression Models Predicting Facebook Use and Facebook Network Characteristics from Personality Factors and Demographic Variables. Table Shows Standardised Coefficients (β) and Standard Errors.

Outcome Variable	Facebook time	Facebook Everyday	Network Size	Log Network Size	Network Density	Number of Clusters
Predictor variables						
Emotionality				0.124 (0.086)		
Extraversion	0.143 (0.090)	0.119 (0.093)	0.336** (0.086)	0.396** (0.084)	−0.115 (0.091)	0.047 (0.084)
Openness	−0.241 (0.091)**					
Network size^a^					−0.354** (0.095)	0.571** (0.088)
Gender (male-- > female)		0.073 (0.096)				−0.146^†^ (0.082)
Age	−0.219* (0.097)	−0.352** (0.104)	−0.330** (0.092)	−0.332** (0.085)	−0.468** (0.087)	0.314** (0.085)
Nationality (Dutch-- > other)	0.289** (0.096)	0.191^†^ (0.100)	0.228* (0.092)			
Relationship status (not in a relationship-- > relationship)	−0.175^†^ (0.089)	0.052 (0.092)	0.125 (0.085)	0.061 (0.082)	0.026 (0.084)	−0.144^†^ (0.078)
*R*^2^	0.213	0.157	0.266	0.391	0.283	0.389
Adjusted *R*^2^	0.174	0.116	0.238	0.292	0.255	0.359
Residual std. Error	0.904 (df = 102)	0.936 (df = 102)	0.869 (df = 103)	0.837 (df = 103)	0.859 (df = 103)	0.797 (df = 102)
F statistic	5.514^**^ (df = 5, 102)	3.798** (df = 5, 102)	9.342** (df 4, 103)	12.058** (df = 4, 103)	10.158** (df = 4, 103)	12.998** (df = 5, 102)

Notes: **p < .01. *p < .05. †*p* < .10.

^a^Network size was only included in the models for density and number of clusters.

Extraversion had a marginally significant positive relationship on participants stating that Facebook was part of their everyday activity (ESM Table 2). However, the association between extraversion and Facebook everyday was no longer significant (β = .12, *p* = .20) after controlling for age (Models 3–6). No other personality variables significantly related to everyday use of Facebook (all *p* > .18). The final model was significant (*F* (5, 102) = 3.80, *p* < .01) and accounted for 12% of the variance in everyday use of Facebook (*R*^2^ = .16, adjusted *R*^2^ = .12 . Table 2).

### Personality and Facebook Network Variables

**Figure 2.**
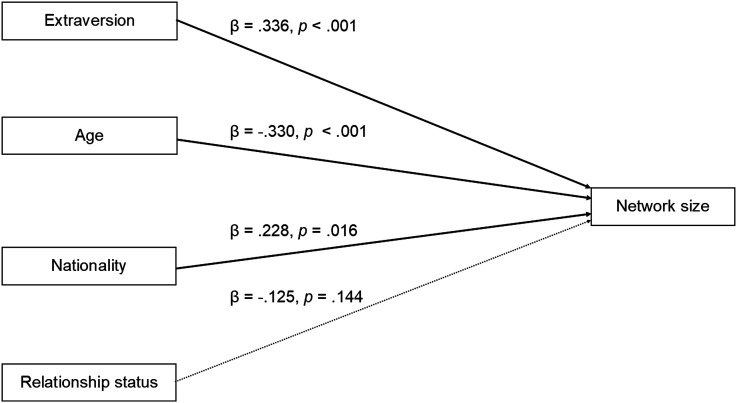
Summary of regression predicting objectively measured Facebook network size from personality and demographic variables. Figure shows final model (*F* (4, 103) = 9.34, *p* < .01, adjusted *R*^2^ = .24) Significant predictors of Facebook time (Extraversion, Age, Nationality) indicated with solid lines; non-significant predictor of Facebook time (Relationship status) indicated with dashed lines. Standardised coefficients (*β*) and *p* values displayed for each predictor. Nationality (0 = Dutch. 1 = Other nationality). Relationship status (0 = Not in a relationship, 1 = In a relationship).

Extraversion was positively related to Facebook network size, supporting our hypothesis 1(c) (ESM Table 3) and this remained the case after the inclusion of the control variables in the regression model (β = .34, *p* < .01; Table 2). No other personality variables significantly related to network size (Model 1, all *p* > .13). The final model was significant (*F* (4, 103) = 9.34, *p* < .01) and accounted for 24% of the variance in Facebook network size (*R*^2^ = .27, adjusted *R*^2^ = .24 Model 4, Figure 2).

**Table 3.** Summary of Hypothesis and Results. Table Shows Statistically Significant Associations (*p* < 0.05) between Variables in Regression Models after Controlling for Demographic Factors (gender, age, nationality, relationship status). Positive Association Indicated by +, negative association indicated by -. No Statistically Significant Association between Variables where Specific Hypotheses were made indicated by NS. Shaded Cells indicate Support for Specific Hypothesis. Hypotheses Numbers are in Brackets.

	FB Time	FB Every day	Network Size	Network Density	Number of Clusters
Extraversion	NS (H1a)	NS (H1b)	+ (H1c)	NS (H1d)	NS (H1e)
Openness to experience	-			NS (H2a)	NS (H2b)
Agreeableness			NS (H3a)	NS (H3b)	NS (H3c)
Conscientiousness	NS (H4a)	NS (H4b)	NS (H4c)	NS (H4d)	
Emotionality			NS (H5a)	NS (H5b)	NS (H5c)
Honesty-humility			NS (H6a)

The results for the log transformation of network size are similar, with a significant positive association between extraversion and log network size (β = .40, *p* < .01; ESM). The final model was significant (*F* (4, 103) = 12.06, *p* < .01) and accounted for 29% of the variance in log transformed network size (*R*^2^ = .39, adjusted *R*^2^ = .29; Table 2).

With regards to network density, initial results showed that Extraversion was negatively related to Facebook network density (Table 2, ESM). No other personality variables were related to network density (all *p* > .14). However, larger networks were significantly less dense and the association between Extraversion and density was no longer significant after controlling for network size (β = −.11, *p* = .20). The final model was significant (*F* (4, 103) = 10.16, *p* < .01) and accounted for 26% of the variance in Facebook network density (*R*^2^ = .28, adjusted *R*^2^ = .26; Model 4).

Finally, with respect to the number of Facebook clusters, Extraversion was positively related to number of clusters, such that more Extraverted users had a larger number of clusters, consistent with hypothesis 1(e). No other personality variables were related (all *p* > .23). However, this effect of extraversion, was no longer statistically significant (β = .05, *p* = .51) after controlling for network size. The final model was significant (*F* (5, 102) = 13.00, *p* < .01) and accounted for 36% of the variance in number of Facebook clusters (*R*^2^ = .39, Adjusted *R*^2^ = .36; Model 7). A summary of the results and how they relate to the hypotheses is provided in Table 3. Overall, of the directional hypotheses, only Hypothesis H1c was supported – users of Facebook who were high in extraversion had a larger Facebook network (i.e. more Facebook Friends).

## Discussion

### Main Findings and Relation to Previous Literature

This study examined the association between the six HEXACO personality factors, Facebook use and objectively measured Facebook network characteristics. Consistent with previous research, our findings suggest that personality variables may be associated with some aspects of Facebook use and characteristics of Facebook networks. Specifically, Extraverts had a larger network size (more Facebook Friends). According to some common estimates of effect sizes (e.g. Fey et al., 2022) the effect size of the association between extraversion and network size was medium, with a standardised coefficient of 0.33, and 26.6% of variance in network size explained by the regression model. Extraverts also had less dense networks - fewer connections between their Facebook Friends. However, this effect was no longer statistically significant after controlling for network size, replicating the finding of Lönnqvist et al. (2014). Our findings demonstrate that, after adjusting for network size, extraverts do not have less dense Facebook networks than introverts, nor do they have more clustering in their networks.

Overall, these results support research that has identified extraversion as the personality factor most consistently associated with the size of both offline (Pollet et al., 2011; Selden & Goodie, 2018) and online social networks (Amichai-Hamburger & Vinitzky, 2010; Bowden-Green et al., 2020; Lönnqvist et al., 2014; Noë et al., 2016). This finding supports the rich-get-richer hypothesis whereby the sociable nature of extraverts means they benefit the most from the opportunities for socializing online (Valkenburg & Peter, 2009). Specifically, as the situation activation mechanism (De Vries et al., 2016) suggests, extraverts seek out situations that fit their personality trait and thus in situations that offer the opportunity for social interaction, extraverts seek social attention (Ashton et al., 2002). Having a larger number of Facebook Friends may be one way extraverts attain social attention, as a larger network means more people to interact with and respond to their posts. Therefore, the affordances offered by Facebook may be especially well suited to extraverted users (Bowden-Green et al., 2020). Future research could focus on how extraversion relates to possible trade-offs between the size of networks and emotional closeness to network members at the different layers of offline and online networks (Dunbar, 2018; Pollet et al., 2011; Sutcliffe et al., 2018; Wagner et al., 2014; Zhu et al., 2013).

Of the other five HEXACO personality traits, those high on Openness to Experience spent significantly less time on Facebook. The standardised coefficient for the association between Openness and time on Facebook was −0.24, again suggesting a medium effect size (Fey et al., 2022), with the overall regression model explaining 21.3% of variance in time spent on Facebook. These findings contradict earlier studies, which found *positive* associations between Openness to Experience and Facebook use (Amichai-Hamburger & Vinitzky, 2010; Gosling et al., 2011; Ross et al., 2009). One reason for this difference in findings could be that Facebook is no longer viewed as a novel platform of online interaction, as it first became available to the public in 2006 (Phillips, 2007). Whist the overall number of Facebook users continue to grow (Dixon, 2022a), it is becoming less popular with users under the age of 25, who are turning to newer social networking platforms such as Twitter, Instagram and TikTok (Auxier & Anderson, 2021; Dixon, 2022b). Those who are high on Openness to Experience are inquisitive and seek novel domains and unusual ideas (Ashton & Lee, 2007). As such, they seek out and create situations where they can express these traits (De Vries et al., 2016), such as newer social media sites. Future research could therefore examine whether those high on Openness to Experience are particularly more likely to spend less time on Facebook in favour of newer social networking sites and messaging services.

Contrary to our hypotheses, the other HEXACO personality characteristics of Agreeableness, Conscientiousness, Emotionality and Honesty-Humility were not significantly associated with Facebook use or Facebook network characteristics. This contrasts with research which has found associations between these or related personality characteristics in both offline (Molho et al., 2016; Wagner et al., 2014) and online (Gosling et al., 2011; Lönnqvist et al., 2014; Noë et al., 2016) social networks. Most of these studies use the five-factor model of personality, as well as different sets of control variables, making it difficult to directly compare the results across studies. Further, whilst Facebook is primarily used to maintain existing relationships (Burke & Kraut, 2016), how the variation in offline sociality relates to the properties of online social networks is still unclear (Sutcliffe et al., 2018, 2022). Future research could examine whether these personality factors may have more influence in different layers of the online network (Molho et al., 2016) or emotional closeness to network members (Pollet et al., 2011; Wagner et al., 2014), rather than global properties of online networks.

Taken as a whole, the findings of this study and previous research suggest that Extraversion is the personality trait most consistently associated with higher levels of social media activity across multiple social media platforms (Bowden-Green et al., 2020). Both on Facebook (this study, Amichai-Hamburger & Vinitzky, 2010; Shen et al., 2015) and other social media platforms including Instagram (Peterka-Bonetta et al., 2021), Twitter (Peterka-Bonetta et al., 2021) and TikTok (Meng & Leung, 2021), extraverted people take advantage of the affordances of these social media sites to build larger networks, interact frequently with other users and thereby gain social attention from others (Ashton et al., 2002). There is consistency in findings both for studies based on self-report data (e.g. Simoncic et al., 2014) and also studies based on objective measurements of social media use (e.g. Azucar et al., 2018; Peterka-Bonetta et al., 2021), suggesting that the associations between extraversion and social media activity are not due to the unreliability of self-reported social media use, in terms of extraverts overestimating their social media activity (Parry et al., 2021). These consistent results for extraversion (Azucar et al., 2018; Bowden-Green et al., 2020) are in contrast to the very inconsistent results for associations between social media use and mental health or well-being (e.g. Coyne et al., 2020; Orben, 2020; O’Day and Heimberg, 2021; Valkenburg et al., 2022), suggesting that it may be easier to determine reliable predictors of social media use such as extraversion than the consequences of social media use.

In addition to the analysis of how personality traits related to Facebook usage and Facebook network characteristics, we also examined how the demographic characteristics of the participants were associated with these variables. Younger participants were more likely to agree that they used Facebook every day, had a larger network size, a correspondingly lower network density, and a larger number of clusters of closely connected friends. These findings are consistent with previous research reporting more intensive Facebook use in younger participants (McAndrew & Jeong, 2012; Ozimek & Bierhoff, 2016) and extend these findings, as they are based an objective measure of the number of Facebook Friends rather than relying on self-report. There was also an effect of gender, with male participants having more tightly connected clusters of friends than females, despite no significant differences in network size between males and females. This is consistent with previous research on gender differences in friendship styles based on social media profile pictures (David-Barrett et al., 2015) which suggested that whilst females prefer dyadic relationships which would lead to fewer clusters, men prefer larger, interconnected friendship groupings which would lead to more clusters.

### Limitations and Future Research

There were several limitations of this study which could be addressed in future research. First, our sample consisted exclusively of university students. Younger participants in this study reported using Facebook more often and had larger, less dense networks, showing the characteristics of the sample can influence the properties of Facebook networks. As such, this sample does not provide an adequate representation of the diverse population of all Facebook users (Auxier & Anderson, 2021; Henrich et al., 2010). Future work could explore whether the relationship between personality and Facebook is affected by broader cultural differences (e.g. Eşkisu et al., 2017; Qiu et al., 2013). Second, the sample size of this study was limited by the fact that participants had to come into the lab to complete the study and agree to have their Facebook network data extracted, rather than just completing an online questionnaire. Whilst the study was adequately powered to detect a weak to moderate effect size, caution should be used in interpreting the findings as conclusively showing positive, negative or no associations between personality factors and Facebook networks and use. Third, whilst the Facebook network characteristics (size, density, clusters) were based on objective measurements, we relied on self-report for the time spent on Facebook and whether participants viewed Facebook as part of their everyday activity. There is only a moderate correlation between subjective reports of time spent on social media and objective data (Parry et al., 2021), and future research could use objective measurements of social media usage and activity to examine how personality characteristics are associated with time spent on social media (e.g. Johannes et al., 2021). Fourth, due the functionality of the *GetNet* app, we were only able to extract data on the properties of the participants' Facebook networks, not the participants' activity on Facebook, for example the frequency and type of posting, commenting and private messaging. Examination of this more detailed usage data may have revealed other associations between personality traits such as Openness to Experience and Conscientiousness, as in previous research (Amichai-Hamburger & Vinitzky, 2010). Finally, this study was limited to Facebook and given the declining use of Facebook amongst younger age groups (Auxier & Anderson, 2021) future studies could examine how personality factors are associated with a broader range of social networking sites such as Instagram, Twitter and TikTok (Bowden-Green et al., 2020; Hughes et al., 2012; Moore & Craciun, 2021; Stokes et al., 2016; Stokes, 1985).

## Conclusion

Overall, two personality factors were associated with Facebook use and Facebook networks. Extraverts had a larger number of Facebook Friends and those high on Openness to Experience reported using Facebook for a shorter duration of time. However, Honesty-Humility, Emotionality, Agreeableness and Conscientiousness were not significantly related to Facebook use, or the characteristics of Facebook network, suggesting that the associations between personality and the Facebook use and Facebook networks considered in this study may be limited to specific personality traits. These findings suggest that just as personality traits such as Extraversion are a significant influence on the size and structure of ‘offline’ social networks (Selden & Goodie, 2018; Wagner et al., 2014), some personality traits may also influence sociality on Facebook (Bowden-Green et al., 2020). Facebook is primarily used to maintain existing social relationships (Burke & Kraut, 2016; Ellison et al., 2014; Sutcliffe et al., 2022) and as such may reflect associations between personality and ‘offline’ networks (Pollet et al., 2011; Selden & Goodie, 2018). Further research can explore how personality traits relate to other social networking sites such as Instagram, Twitter (Peterka-Bonetta et al., 2021) and TikTok (Meng & Leung, 2021), through which users connect to both people they know offline and a wide range of other users they do not know personally, such as celebrities and influencers (Auxier & Anderson, 2021).

## Notes

1. We use a capital F when referring to Facebook Friends, to distinguish this from friendship in the more general sense. In Facebook, users can send a Friend request to other users and this request must be accepted by the other user for the two users to be able to be connected as Friends and view each other’s profiles.

2. Facebook time and Facebook everyday were treated as continuous variables for the main analyses. The Electronic Supplementary Material (ESM) contains supplementary analyses where we treated Facebook time and Facebook everyday as ordinal variables (https://osf.io/4kjfp/). These analyses produced the same key findings as the main analyses.
